# Psychometric Properties of the Adverse Childhood Experiences Abuse Short Form (ACE-ASF) for Ecuadorian Youth

**DOI:** 10.3390/ejihpe15040063

**Published:** 2025-04-16

**Authors:** Andrés Ramírez, Luis Burgos-Benavides, Jessica Vanessa Quito-Calle, Hugo Sinchi-Sinchi, Javier Herrero Díez, Francisco Javier Rodríguez-Díaz

**Affiliations:** 1 Department of Clinical Psychology, Universidad Politécnica Salesiana, Cuenca 010107, Ecuador; jquito@ups.edu.ec; 2 Department of Psychology, Universidad de Oviedo, 33003 Oviedo, Spain; burgosluis@uniovi.es (L.B.-B.); herrero@uniovi.es (J.H.D.); gallego@uniovi.es (F.J.R.-D.); 3 Department of Psychology, Pontificia Universidad Católica del Ecuador, Esmeraldas 080101, Ecuador; hfsinchi@pucese.edu.ec

**Keywords:** psychometrics, child physical abuse, child sexual abuse, validation

## Abstract

Adverse childhood experiences, such as abuse, are a risk factor for mental health and poor socio-emotional development in adulthood. Assessing these experiences in specific populations allows for the identification of patterns and the implementation of preventive interventions. Objective: To evaluate the psychometric properties of the abbreviated version of the Adverse Childhood Experiences Abuse Form (ACE-ASF) in Ecuadorian youth, aiming to ensure the validity, reliability, and consistency of the instrument in accurately measuring abuse dimensions in this Ecuadorian population. Methodology: An instrumental study was conducted on the psychometric properties of the eight-item ACE-ASF, applying it to a sample of 840 university students (52.1% females and 47.9% males). The evaluation focused on analyzing the factorial structure and internal consistency of the instrument in this sample. Results: The two-factor model showed a satisfactory fit across all levels of invariance (configural, metric, scalar, and strict), with acceptable fit indices (CFI, TLI, GFI, RMSEA, and SRMR). The internal consistency was adequate, as assessed using the McDonald’s omega and Cronbach’s alpha coefficients. Convergent and discriminant validity were confirmed using the AVE and HTMT indices, ensuring proper differentiation between the dimensions assessed. Conclusion: The ACE-ASF proved to be a valid and reliable instrument for assessing abuse experiences in Ecuadorian youth. Its two-factor structure reflects distinct yet related dimensions, providing a useful tool for identifying adverse childhood experiences in this population.

## 1. Introduction

Violence against children is a critical public health issue, affecting nearly one billion children annually (Ferrara et al., 2024; Hillis et al., 2016; Thomas, 2024). Adverse childhood experiences (ACEs), including physical, emotional, and sexual abuse, as well as neglect, are linked to a range of long-term consequences that extend beyond childhood and into adulthood. These experiences significantly impact physical, mental, and reproductive health, contributing to chronic conditions such as cardiovascular disease, obesity, and diabetes (Ali et al., 2024; Güler et al., 2024; Swift et al., 2024). Furthermore, ACEs affect cognitive and social development, resulting in poor academic performance, impaired emotional regulation, and difficulties in forming and maintaining healthy relationships during childhood (Bhutta et al., 2023; Qu et al., 2024). Adolescents and young adults (over 18 years) who experienced ACEs are also more likely to face mental health challenges, including depression, anxiety, and post-traumatic stress disorder (PTSD) (Horino et al., 2023).

The neurobiological effects of ACEs are profound, disrupting brain development and affecting stress response systems, emotional regulation, and decision-making processes. These disruptions persist in adulthood, affecting emotional and cognitive functioning, which can impact various aspects of life, such as academic success, work performance, and interpersonal relationships (Jewkes et al., 2010). Adolescents and young adults who have been exposed to childhood violence are also more likely to engage in high-risk behaviors such as smoking, alcohol and drug use, and risky sexual practices. These behaviors are strongly associated with the development of non-communicable diseases, cancer, and sexually transmitted infections in both adolescents and adults (Meinck et al., 2023; Miller et al., 2011; Ramiro et al., 2010; Sansone et al., 2022).

In addition to the profound individual health consequences, adverse childhood experiences (ACEs) play a significant role in perpetuating cycles of violence that extend far beyond childhood. Adolescents and young adults who have endured abuse during their early years face an increased risk of becoming either victims or perpetrators of violence in later stages of life (Butler et al., 2020). This cyclical pattern not only affects their personal well-being but also contributes to the intergenerational transmission of abuse, reinforcing a social and familial environment where violence becomes normalized. Moreover, the long-term psychological, emotional, and behavioral effects of ACEs can lead to difficulties in forming healthy relationships, poor emotional regulation, and an increased likelihood of engaging in high-risk behaviors, further exacerbating the cycle of violence. Addressing these adverse experiences through early intervention, trauma-informed care, and supportive social policies is essential for breaking this harmful pattern and fostering healthier, safer communities (Herrenkohl et al., 2022; Marshall et al., 2022; Shields et al., 2020). These patterns underscore the critical need for early intervention strategies that focus not only on children but also on young adults who have experienced ACEs. Addressing these experiences in both childhood and adulthood can prevent the long-term effects of abuse, break the cycle of violence, and promote resilience and recovery in individuals. The development of culturally sensitive, evidence-based interventions that target both children and young adults is essential for mitigating the lasting impacts of ACEs and promoting healthier, more resilient individuals and communities.

### 1.1. Methodological Approaches to Studying Childhood Violence and Adverse Childhood Experiences

Research on childhood violence and adverse childhood experiences (ACEs) has employed a range of methodological approaches aimed at understanding the prevalence, long-term impact, and diverse outcomes of abuse. Large-scale cross-sectional studies, such as the Violence Against Children Surveys (Centers for Disease Control and Prevention, 2015) and the Optimus Studies (UBS Optimus Foundation, 2015), primarily focus on assessing the incidence of childhood victimization, identifying perpetrators, and exploring a limited set of outcomes related to the immediate effects of abuse. These studies predominantly concentrate on children under 18 years of age, providing valuable data on the scope and nature of abuse in this age group (May-Chahal & Cawson, 2005; May-Chahal et al., 2006).

In addition to these studies, repeated cross-sectional studies, such as those conducted by the UK’s National Society for the Prevention of Cruelty to Children (NSPCC), allows for the examination of trends in the prevalence of child abuse over time. By tracking these trends, researchers can identify whether rates of abuse are increasing, decreasing, or fluctuating, providing critical insights into the effectiveness of current prevention strategies and policy interventions (May-Chahal & Cawson, 2005; May-Chahal et al., 2006). This type of data is essential for recognizing shifts in societal and cultural attitudes toward child abuse and the evolving risk factors contributing to its occurrence.

Longitudinal studies, such as the LONGSCAN Consortium, are particularly valuable, as they track cohorts of children over extended periods, sometimes decades, to understand the long-term evolution of abuse and its lasting effects on victims. These studies help to map how childhood violence can shape mental health outcomes, behavioral issues, and overall well-being, extending well into adolescence and even into young adulthood, often beyond the age of 18. The ability to track these individuals over time also allows for a deeper understanding of the cumulative impact of multiple ACEs on a person’s life trajectory, including their adult health, socioeconomic status, and interpersonal relationships (Fry et al., 2012).

Moreover, multi-country health surveys, which incorporate child abuse assessments alongside other health-related topics, offer a global perspective on the prevalence and impact of childhood violence. These studies enable comparisons across various regions, revealing differences and similarities in how child abuse is experienced and addressed across cultures, healthcare systems, and legal frameworks (Meinck et al., 2017, 2020; Steele et al., 2024). Such comparative data are crucial for identifying universal patterns of abuse and understanding region-specific challenges, particularly in low- and middle-income countries where child protection services may be less developed.

Despite the valuable insights offered by these methodologies, significant challenges persist in validating and ensuring the reliability of measures used to assess child abuse, particularly in diverse cultural contexts. Different cultural perceptions of what constitutes abuse, as well as varying levels of willingness to report it, can introduce biases in data collection. For example, behaviors that are considered abusive in one culture may be viewed as acceptable or even necessary in another. This complicates the development of universally applicable definitions and instruments for assessing child abuse and ACEs, which in turn affects the accuracy of the data collected.

In addition, the methodologies used in child abuse research often face issues related to the underreporting of abuse, either due to fear of repercussions or due to a lack of awareness about what constitutes abuse. Ensuring that assessment tools are culturally sensitive, reliable, and valid across different populations is critical for producing accurate data. Furthermore, longitudinal studies that track individuals into adulthood are especially important in understanding how early experiences of violence and neglect contribute to ongoing mental health challenges, substance abuse, and patterns of re-victimization or perpetration of violence in later life.

The need for accurate and culturally sensitive tools is underscored by the growing interest in studying the psychometric properties of instruments like the Adverse Childhood Experiences Abuse Short Form (ACE-ASF) in specific populations, such as Ecuadorian youth. The ACE-ASF, designed to assess the extent of childhood abuse, requires thorough validation in local contexts to ensure its effectiveness and reliability. This process involves examining its internal consistency, construct validity, and applicability to Ecuador’s cultural and socio-economic environment, where unique challenges in child protection and reporting practices may affect the instrument’s performance. Addressing these limitations is crucial for enhancing the accuracy of childhood violence assessments and informing the development of more effective intervention strategies. Improvements in measurement tools, better cultural adaptation of research instruments, and more inclusive data collection strategies are all necessary steps to improve the overall quality of research on childhood violence and adverse childhood experiences (ACEs). Ultimately, this will lead to more effective interventions that not only address immediate issues but also promote long-term healing and resilience among victims while reducing the overall burden of abuse on society.

Studies on childhood violence have mainly focused on high-income countries, leaving a significant gap in regards to the Ecuadorian context. No prior research has analyzed this phenomenon using validated instruments in the local population, making it difficult to develop prevention and intervention strategies tailored to the country’s realities. The lack of specific data limits the understanding of the sociocultural and economic factors that influence the identification, reporting, and management of childhood violence in Ecuador. Therefore, it is essential to conduct assessments that take these factors into account, allowing for the collection of accurate information to enhance the effectiveness of public policies and child protection programs.

### 1.2. Adverse Childhood Experiences Questionnaire

The ACE is one of the most widely used instruments for evaluating childhood trauma and violence. It is designed to assess various domains of early-life adversities, including physical, emotional, and sexual abuse, neglect, and household dysfunction. These domains collectively capture the broad range of stressful and harmful experiences that can shape a child’s psychological and physiological development, with lasting effects on health throughout the lifespan. By identifying these risk factors early, the ACE Questionnaire serves as a powerful tool for understanding the long-term consequences of childhood adversity and informs preventive and intervention strategies (Felitti et al., 1998; Hulme, 2007; Liebschutz et al., 2018; Midei et al., 2013). Initially developed for use in high-income countries, its application has expanded significantly in low- and middle-income countries, underscoring the universal nature of childhood trauma and the increasing recognition of its global prevalence (Mikton et al., 2013). This global expansion has prompted adaptations of the instrument to account for different sociocultural contexts, enhancing its relevance for a diverse range of populations.

The ACE Questionnaire is constructed on a solid psychometric foundation, demonstrating a reliable three-factor structure that captures physical/emotional abuse, household dysfunction, and sexual victimization. These factors have been shown to correlate with various adverse health outcomes, including mental health disorders, substance abuse, and chronic diseases. The tool’s construct validity is well-established, meaning it accurately measures what it intends to, and its internal consistency is strong, reflecting the robustness of the instrument in capturing various dimensions of early-life trauma. The reliability of the ACE Questionnaire makes it a valuable resource for both research and clinical purposes, as it consistently demonstrates its ability to predict negative health outcomes across diverse populations (D. C. Ford et al., 2014; K. Ford et al., 2020; Holden et al., 2020; Wingenfeld et al., 2011; Kovács-Tóth et al., 2023). Its wide use in research has made it a cornerstone in the study of how childhood adversity impacts adult health and well-being.

Recognizing the need for a more culturally adaptable version, the World Health Organization (WHO) developed the ACE-International Questionnaire (ACE-IQ), a 43-item instrument tailored for use in diverse cultural settings, including low- and middle-income countries. The ACE-IQ was specifically designed to retain the core domains of the original ACE Questionnaire, while incorporating relevant cultural nuances that might affect how childhood trauma is experienced and reported in different societies. This version has broadened the scope of ACE research, allowing for more inclusive assessments of childhood adversity across the globe (World Health Organization, 2020). The ACE-IQ has been implemented in various countries, including Kenya (Goodman et al., 2017), Brazil (Soares et al., 2016), and Iraq (Almuneef et al., 2014), with preliminary studies supporting its usefulness in these diverse settings. However, the need remains for further investigation into the psychometric properties of the ACE-IQ in these regions, as limited data on its reliability and validity persist. These gaps suggest the necessity of more cross-cultural research to ensure the instrument’s accuracy and to better understand its applicability in different cultural and socioeconomic contexts (Pace et al., 2022; Tran et al., 2015).

In addition to the full ACE and ACE-IQ versions, the ACE Abuse Short Form (ACE-ASF), an abbreviated eight-item version that specifically targets abuse-related experiences, is also available. This shorter form is particularly useful for situations where time constraints or resource limitations may require a more focused assessment of abuse dimensions. The ACE-ASF is designed to be used with adolescents and adults, offering a more efficient means of gathering critical data on abuse while retaining the core elements of the original ACE Questionnaire. Though promising in its utility, the psychometric validation of the ACE-ASF across diverse populations and cultural settings remains incomplete. As a result, additional research is needed to explore its reliability, construct validity, and effectiveness in capturing the complexities of abuse experiences in different demographic groups. This is especially important in the case of Ecuadorian youth, where understanding the psychometric properties of the ACE-ASF is crucial for ensuring that the instrument accurately reflects the unique sociocultural dynamics and patterns of childhood abuse in the Ecuadorian context. Such validation will help ensure that the ACE-ASF is both a reliable and culturally sensitive tool for assessing childhood abuse in Ecuador, allowing for more effective interventions and support for youth affected by trauma (Meinck et al., 2017).

In general, while the ACE and its adaptations have become indispensable tools for identifying the impact of childhood adversity, there is still much work to be done in refining and validating these instruments across a variety of cultural and socio-economic contexts. The ongoing expansion of ACE research into low- and middle-income countries and the development of tools like the ACE-IQ and ACE-ASF represent significant strides toward understanding and mitigating the long-term effects of childhood trauma. Nevertheless, continuous research and psychometric testing are necessary to further improve the global applicability, reliability, and validity of these essential instruments, ensuring that they can serve as effective tools for prevention, early intervention, and support for individuals affected by childhood violence.

### 1.3. Study Objective

The primary objective of this study is to analyze the psychometric properties of the abbreviated version of the Adverse Childhood Experiences Questionnaire (ACE-ASF) in Ecuadorian youth, with a particular focus on ensuring its reliability, validity, and measurement invariance. This evaluation aims to confirm that the instrument is suitable for accurately measuring the dimensions of abuse within this specific population, ensuring that it consistently captures the intended constructs.

The first objective is to analyze the reliability of the ACE-ASF in the Ecuadorian youth population to ensure the stability of the instrument. This analysis aims to determine whether the questionnaire maintains a high level of internal consistency when measuring adverse experiences, which is essential for its application in future research. The second objective is to analyze the structural validity of the ACE-ASF through confirmatory factor analysis (CFA), with the aim of evaluating whether the dimensions of the questionnaire, such as physical–emotional abuse and sexual abuse, are grouped according to the expected theoretical structure, ensuring that the model is valid and suitable for the Ecuadorian youth population. The third objective is to evaluate the convergent and discriminant validity of the ACE-ASF to verify that the abuse dimensions are measured accurately and distinctly, ensuring that each category of the questionnaire adequately captures the different types of adverse experiences. Finally, the fourth objective is to examine the invariance of the ACE-ASF model by sex, with the aim of ensuring that the instrument is equally applicable and valid for both males and females, avoiding gender-related biases among participants.

The hypotheses are as follows: The first hypothesis is that the ACE-ASF presents high reliability in the Ecuadorian youth population, as evidenced by a significant level of internal consistency in measuring adverse experiences. The second hypothesis is that the structure of the ACE-ASF adequately fits the underlying theory, with the dimensions of physical–emotional abuse and sexual abuse grouping coherently according to the proposed theoretical model, as demonstrated by the Confirmatory Factor Analysis (CFA). The third hypothesis is that the dimensions of physical–emotional abuse and sexual abuse in the ACE-ASF show significant convergent and discriminant validity, ensuring that each category of the questionnaire accurately and distinctly measures the different types of adverse experiences. Finally, the fourth hypothesis is that the ACE-ASF model is invariant by sex, meaning the instrument is equally applicable and valid for both males and females, without gender-related biases.

## 2. Materials and Methods

### 2.1. Research Design

Instrumental research was conducted in two phases, following the current standards for the validation of educational and psychological tests (Arias & Sireci, 2021), as well as the guidelines for the adaptation and translation of existing tests (Brauer et al., 2023; Cruchinho et al., 2024; Muñiz et al., 2013; Moore et al., 2021). In the first phase, linguistic adaptation of the original test was carried out (Meinck et al., 2017) through an iterative translation process performed by experts (Elosua et al., 2014). In the second phase, evidence of the reliability and validity of the instrument was collected.

### 2.2. Participants

The sample included a total of 840 participants selected by means of a non-probabilistic convenience sample (Teodoro Dantas Sartori, 2024). In terms of sex, the majority of participants identified themselves as females (52.1%, *n* = 438), while 47.9% (*n* = 402) identified themselves as males. In terms of ethnicity, the vast majority of participants (90.2%, *n* = 758) identified as Mestizo. Other ethnicities were represented to a lesser extent: 6.3% of the participants (*n* = 53) identified themselves as Afro-Ecuadorian, 1.5% as White (*n* = 13), 1.2% as Montubio (*n* = 10), and 0.7% as Indigenous (*n* = 6). Regarding the age of the participants, the analysis yielded a mean of 20.61 years, with a standard deviation of 2.52, and a median of 21 years, which suggests a slight concentration of ages around this value. The participants were selected through a non-probabilistic convenience sampling method, with inclusion criteria requiring participants to be active university students, aged 18 or older, and to participate voluntarily. Exclusion criteria included individuals who did not meet these requirements or failed to complete the evaluation instruments properly. Statistical analyses were conducted using SPSS version 26, and descriptive tests, including determining the mean, standard deviation, and median, were applied to describe the sample characteristics.

### 2.3. Measures

#### 2.3.1. Ad Hoc Sociodemographic Survey

An ad hoc sociodemographic data survey was administered to collect the age, sex, and ethnicity of the participants.

#### 2.3.2. Adverse Childhood Experiences Abuse Short Form (ACE-ASF)

The form consists of eight Likert-type items. In the exploratory factor analysis in the research of Meinck et al., 2017, a two-factor structure was established, comprising physical–emotional abuse and sexual abuse. In the confirmatory factor analysis, the study consluded that this model fit the data well [*χ*^2^(*gl*) = 60.526(19); *RMSEA* = 0.036; *CFI/TLI* = 0.990/0.986]. Metric invariance between sexes was supported. Internal consistency was good (*α* = 0.83) for the sexual abuse scale and low (*α* = 0.57) for the physical–emotional abuse scale (Meinck et al., 2017).

### 2.4. Procedure

The study was conducted following the ethical guidelines of the Declaration of Helsinki. The research was approved by the Human Research Ethics Committee of the Pontificia Universidad Católica del Ecuador under Protocol *No PV-14-2022*. Data collection was performed through the application of face-to-face surveys, where participants completed the self-report form using a pencil and paper.

After obtaining the necessary permissions from the educational institutions, the participants were invited to take part in the study. They were informed of the purpose and importance of the research, emphasizing the voluntary nature of their participation. Additionally, they were informed of their right to withdraw or change their minds at any time during the process. Each participant signed an informed consent form, ensuring that they understood and accepted the terms of their participation.

Given the sensitive nature of the topics related to adverse childhood experiences (ACEs), additional ethical safeguards were implemented. The study received approval from the corresponding institutional ethics committee, which ensured adherence to ethical guidelines for research involving vulnerable populations. Confidentiality and data protection measures were strictly maintained, following national and international ethical standards. Participants were also provided with psychological support resources in case the discussion of ACEs triggered emotional distress. Furthermore, the research team was trained in trauma-sensitive approaches to minimize any potential harm and ensure a safe environment for all participants.

The administration of the instruments (a three-item sociodemographic survey and the eight-item Adverse Childhood Experiences Abuse Short Form) had an average duration of seven minutes per participant. Each participant was assigned a unique code to ensure data anonymization.

The responses obtained were transcribed into the *OpenClinic* data management system, where they were analyzed in regards to several aspects: (a) sociodemographic characteristics, (b) perception of conflict resolution style by dimensions, (c) conflict resolution style according to sex, and (d) confirmatory factor analysis. First, descriptive analyses were carried out for each item and for the general dimensions of childhood grandparenting. Measures of central tendency (mean and median) and dispersion (standard deviation) were calculated for each item, as well as skewness and kurtosis to evaluate the distribution of responses. These analyses revealed patterns of responses among the young people and allowed for the identification of items with high skewness and kurtosis, indicating less common adverse experiences in the sample.

### 2.5. Statistical Analysis

The analyses were performed using *R* version 4.4.3 (R Core Team, 2022) and *JASP* version 0.19.3. Regarding the first objective, to analyze the reliability of the ACE-ASF in the Ecuadorian youth population, item-descriptive statistics such as mean, standard deviation, skewness, and kurtosis were calculated. Normality was assessed using the Shapiro–Wilk test (Demir, 2022; Monter-Pozos & González-Estrada, 2024; (Shapiro & Wilk, 1965). Additionally, reliability estimates (Malkewitz et al., 2023) such as Guttman’s lambda-2 (λ2) and lambda-6 (λ6), as well as the greatest lower bound (GLB), were used to measure the internal consistency of the questionnaire, considering total variance, covariances between items, and residual errors. The average interitem correlation (AIC) was also calculated to assess the average correlation between items. Descriptive measures, including mean, standard deviation, and 95% confidence intervals for each reliability estimate, were reported to evaluate the precision and stability of the results.

Regarding the second objective, to analyze the structural validity of the ACE-ASF through confirmatory factor analysis (CFA), the factor structure of the questionnaire was examined using the *Lavaan* statistical package (Rosseel et al., 2025). Since the data did not meet the assumption of multivariate normality (DiStefano et al., 2021; Shi et al., 2021), the diagonally weighted least squares estimator (DWLS) was applied (Du & Bentler, 2022). Fit indices were evaluated using a comparative fit index (CFI) of 0.95 and a standardized root mean square residual (SRMR) of 0.08, which were established as the optimal model fit criteria (Cho et al., 2020; Hu & Bentler, 1999; Shi & Maydeu-Olivares, 2020). The reliability of the factors was further assessed using Cronbach’s alpha and McDonald’s omega coefficients (Revelle, 2019).

Regarding the third objective, to evaluate the convergent and discriminant validity of the ACE-ASF, two key indicators were used, i.e., the average variance extracted (AVE) and the heterotrait–monotrait ratio (HTMT). These indicators provide evidence of scale validity, with reference values of AVE ≥ 0.500 and HTMT ≤ 0.950 (Cheung & Wang, 2017). The statistical analyses included the composite reliability (CR > 0.70) and variance inflation factor (VIF < 10) (Kalnins & Praitis Hill, 2025). Additionally, Spearman’s correlation coefficient (De Winter et al., 2016) was applied to examine the relationship between the factors of physical–emotional abuse (summation of items A1–A4) and sexual abuse (summation of items A5–A8). This analysis ensured that the dimensions of the questionnaire were measured accurately and distinctly, confirming the validity of each category in capturing different types of adverse experiences.

To address the fourth objective, which involves examining the invariance of the ACE-ASF model by sex, a confirmatory factor analysis (CFA) was conducted to verify the factorial structure of the questionnaire, followed by measurement invariance testing through configural, metric, scalar, and strict invariance models (Hu & Bentler, 1999; Shi et al., 2021). CFA allows for assessing whether the questionnaire items adequately reflect the theoretically established dimensions, while measurement invariance ensures that the instrument measures the same construct equivalently in both males and females. These analyses confirm the validity of the questionnaire for both groups and prevent gender-related biases in the interpretation of the results. Measurement invariance refers to the statistical property that ensures that a test or questionnaire functions the same way across different groups, allowing for meaningful comparisons (Shi & Maydeu-Olivares, 2020).

In future research, it is important to consider how the cultural context of Ecuador might influence the choice between the ACE-ASF (Adverse Childhood Experiences—Adult Survey Form) and the ACE-IQ (Adverse Childhood Experiences—Interview Questionnaire). The sociocultural context of Ecuador, with its specific perceptions of family, abuse, and power dynamics, can affect local researchers’ and healthcare professionals’ preferences. For example, mental health professionals may lean towards one tool over the other, depending on their familiarity with the instrument, their perception of its validity in the Ecuadorian context, and how comfortable individuals feel using each one. Additionally, cultural stigma surrounding certain types of abuse, such as sexual abuse, could influence people’s willingness to acknowledge and report such experiences, impacting the research outcomes and the effectiveness of the assessment tools.

In this regard, a valuable line of inquiry would be to explore how cultural stigma and social norms impact the perception and reporting of adverse experiences, especially in regards to sexual abuse. Conducting qualitative research using in-depth interviews could provide a clearer understanding of how different communities in Ecuador perceive abuse and how these perceptions influence the willingness to report it. Furthermore, strategies could be developed to adapt the measurement tools to the country’s cultural specifics, such as modifying the language used and implementing awareness-raising approaches, in order to improve the accuracy and effectiveness regarding assessing childhood adverse experiences within the Ecuadorian population.

Finally, a network analysis (Borsboom et al., 2021; Christodoulou et al., 2019; Epskamp et al., 2018) was conducted to explore the relationships and centrality of items within the questionnaire. Measures of centrality (betweenness, closeness, strength, and expected influence) and clustering methods (Barrat, Onnela, WS, and Zhang) were employed to provide additional insights into the questionnaire’s structure and the interconnections between its components. These analyses contribute to a comprehensive understanding of the ACE-ASF’s psychometric properties, ensuring its reliability, validity, and applicability for the Ecuadorian youth population.

## 3. Results

Table 1 presents the descriptive data for the items and dimensions of the Adverse Childhood Experiences Abuse Short Form (ACE-ASF) in Ecuadorian youth (*n* = 840). Overall, the individual items showed notable variability, with medians ranging from 2 to 3 and means ranging from 2.07 (A8) to 3.283 (A1). Items A1 and A3 showed distributions closer to symmetry, with lower skewness and kurtosis values, while items A5 to A8, related to sexual abuse experiences, displayed higher levels of skewness and kurtosis, especially item A8, which showed a skewness of 2.259 and a kurtosis of 8.944. In all cases, the Shapiro–Wilk test revealed significance (*p* < 0.001), suggesting that none of the distributions followed a normal pattern.

Regarding the grouped dimensions, dimension F1 (physical and emotional abuse, sum of A1 to A4) showed a median of 10 and a mean of 11.308, with a moderately symmetric distribution (*skewness* = 0.542) and kurtosis close to zero (−0.258), indicating a more uniform distribution. On the other hand, dimension F2 (sexual abuse, sum of A5 to A8) showed a median of 8 and a mean of 8.523, with a more asymmetric and leptokurtic distribution (*skewness* = 1.821, and *kurtosis* = 6.902), showing a concentration of values at the lower end. Both dimensions showed significance in the normality test (*p* < 0.001), confirming distribution patterns that did not follow a normal curve.

In the original model, the fit was excellent, with an *χ*^2^ value of 19.587, 19 degrees of freedom, and an *χ*^2^*/df* ratio of 1.031 (*p* = 0.420), indicating that the model fit the data well. The fit indices were optimal, with CFI, TLI, and GFI values of 0.999, and an RMSEA of 0.006, with a confidence interval ranging from 0.000 to 0.031.

To assess sex invariance, the configural, metric, scalar, and strict models were tested. In each model, the fit indices remained high (*CFI*, *TLI*, and *GFI* = 0.999), and the *RMSEA* remained at 0.000, with confidence intervals between 0.000 and 0.023, reflecting a stable fit across different levels of invariance. The configural invariance test yielded an *χ*^2^ value of 27.058 with 38 degrees of freedom (*p* = 0.907), while the strict model showed an *χ*^2^ value of 49.101 with 58 degrees of freedom (*p* = 0.791), indicating that the model is invariant with respect to sex at the configural, metric, scalar, and strict levels.

The results for reliability and validity (convergent and discriminant) for the sex invariance of the Adverse Childhood Experiences Abuse Short Form in Ecuadorian youth are presented below. Regarding reliability, consistent values of McDonald’s omega (*ω*) and Cronbach’s alpha (α) were observed for both factors and across both sexes at the configural, metric, scalar, and strict levels. In the male group, Factor 1 (physical and emotional Abuse) showed moderate to high reliability, with ω and α values ranging from 0.842 to 0.857, while Factor 2 (sexual abuse) maintained high reliability, with ω ranging from 0.771 to 0.801. In the female group, Factor 1 showed higher reliability values than those for the male group, with *ω* ranging from 0.804 to 0.890, and Factor 2 exhibited high reliability, with ω ranging from 0.880 to 0.907.

In terms of validity, AVE (convergent validity) and HTMT (discriminant validity) values confirmed the adequacy of the model for both factors in each group. At the configural level, Factor 1 had an AVE of 0.588 for males and 0.524 for females, while Factor 2 showed an AVE of 0.227 and 0.458, respectively. At the metric, scalar, and strict levels, these values remained stable, suggesting that both convergent and discriminant validity were satisfactory for both factors and sexes. Thus, the results indicated that the model is valid and reliable for assessing childhood adverse experiences in an invariant manner across males and females.

Table 2 provided the standardized factor loadings, as well as the reliability and validity values for the original model of the Adverse Childhood Experiences Abuse Short Form in a sample of Ecuadorian youth. In Factor 1 (physical and emotional abuse), the factor loadings for indicators A1 to A4 ranged from 0.705 to 0.832, showing high internal consistency for this factor, with a McDonald’s omega (*ω*) value of 0.830 and a Cronbach’s alpha (*α*) of 0.823. The overall reliability for this factor was also high, with a total *ω* of 0.872 and a total α of 0.812, indicating adequate cohesion among the items for this factor. The convergent validity (*AVE*) for Factor 1 was 0.553, confirming that the indicators effectively measured the construct of physical and emotional abuse.

In Factor 2 (sexual abuse), the factor loadings for items A5 to A8 were similarly strong, with values ranging from 0.654 to 0.858, particularly highlighting item A5, with a loading of 0.858. This factor achieved acceptable reliability, with a ω of 0.861 and an α of 0.867. The reliability and validity values indicated that Factor 2 remained internally consistent, with an AVE of 0.637, supporting the convergent validity of the factor. Furthermore, the HTMT index of 0.367 reflected adequate discriminant validity between the two factors. These results collectively suggested that the original model is reliable and valid for assessing childhood adverse experiences within the physical–emotional and sexual abuse factors in the studied sample.

The composite reliability (CR) and the variance inflation factor (VIF) are key indicators for assessing the quality of a measurement model. In our study, the CR values for factors F1 and F2 are 0.83 and 0.85, respectively, indicating the high internal consistency of the items, exceeding the accepted threshold of 0.70. This suggests that the items within each factor reliably measure the same construct. On the other hand, the VIF values for F1 and F2 are 1.42 and 1.56, respectively, indicating low multicollinearity among the items, as they fall below the critical threshold of 10. Taken together, these results strengthen the validity and reliability of the measurement model used to evaluate adverse childhood experiences in Ecuadorian youth.

**Figure 1:**
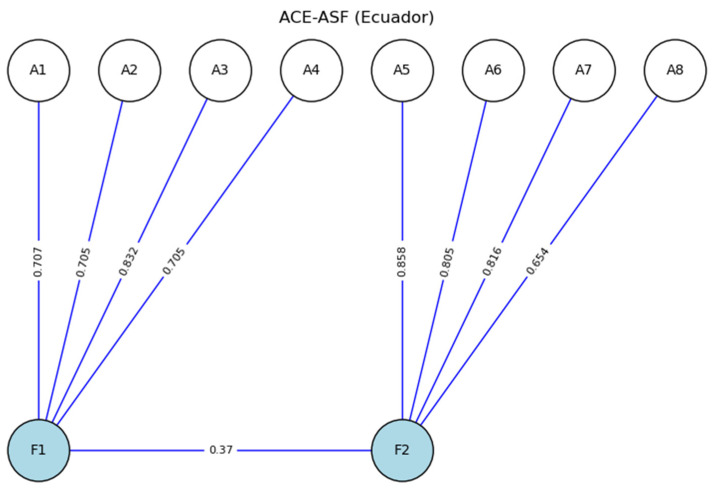
Model of the Adverse Childhood Experiences Abuse Short Form (ACE-ASF). **Note.** F1 = physical and emotional abuse, F2 = sexual abuse, including standardized estimates and correlation between factors. The Python 3.13.3 code can be viewed at the following link (accessed on 1 January 2020): https://n9.cl/5x9ld.

Figure 1 displays the structural model of the Adverse Childhood Experiences Abuse Short Form, consisting of two factors: F1 (physical and emotional abuse) and F2 (sexual abuse). Factor F1 included items A1, A2, A3, and A4, with standardized factor loadings of 0.71, 0.70, 0.83, and 0.70, respectively, with A3 showing the highest association with this factor. Factor F2 comprised items A5, A6, A7, and A8, with loadings of 0.86, 0.80, 0.82, and 0.65, where item A5 showed the highest association with the sexual abuse factor. Additionally, an HTMT index of 0.37 was observed between factors F1 and F2, indicating a moderate correlation between physical–emotional abuse and sexual abuse. The standardized estimates and the HTMT index were presented using two decimal places for greater precision.

A significant correlation of 0.299 (*p* < 0.001) was found between Factor 1 (physical and emotional abuse) and Factor 2 (sexual abuse), indicating a positive, although moderate, relationship between both types of abuse. This correlation suggests that, in this sample, youth who reported experiences of physical and emotional abuse were also moderately likely to have experienced childhood sexual abuse.

**Figure 2:**
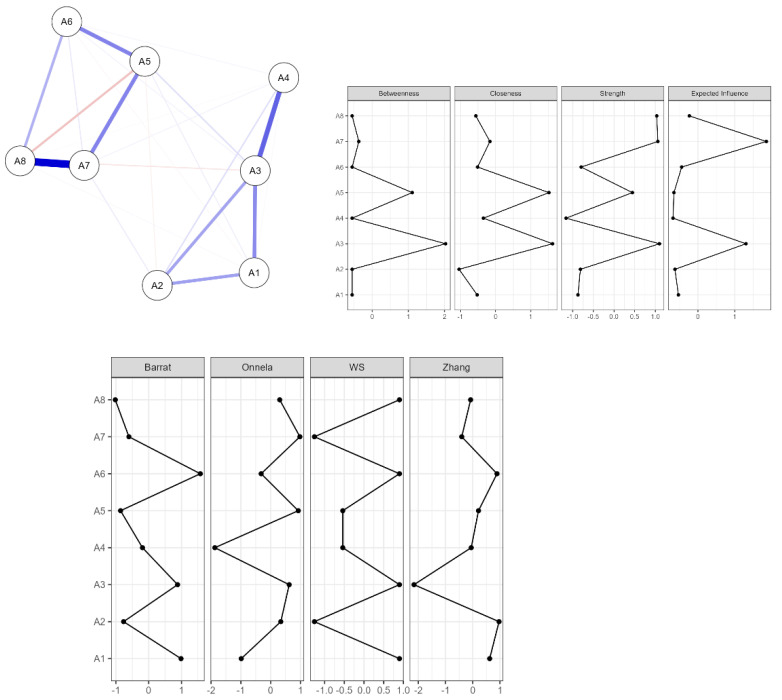
Networks, centrality, and clustering of the Adverse Childhood Experiences Abuse Short Form (ACE-ASF) in Ecuadorian youth.

Figure 2 presented a detailed analysis of the network structure, centrality, and clusters of the items in the Adverse Childhood Experiences Abuse Short Form (ACE-ASF) questionnaire applied to Ecuadorian youth.

In the central panel, the network of interactions between the items (A1–A8) is represented. Each node corresponds to an item from the questionnaire, while the lines connecting them (edges) indicate the magnitude and direction of their relationships. The thickness and color of the lines reflect the intensity of these connections; for example, the interactions between A6 and A7 stand out for their greater strength, represented by thicker and more intensely colored lines. This pattern suggests that these items are strongly related, possibly measuring very similar or complementary aspects within the adverse experience.

The left and right panels present the results of the centrality metrics of the items, such as betweenness, closeness, strength, and expected influence. These metrics identify which items are the most relevant within the network. For example, A7 and A6 show high values across several centrality metrics, indicating that these items are key nodes within the network and play an important role in connecting other items. In contrast, items such as A1 and A4 show lower values, suggesting that they play a more peripheral role in the questionnaire’s structure.

Finally, the cluster analysis identifies groupings of items that share similarities in their relationships. The clusters reflect groups of items that likely assess specific dimensions of adverse experiences. This analysis facilitates the interpretation of the questionnaire by highlighting patterns of relationships among the items, which could be useful for designing more targeted interventions or simplifying the instrument in future applications. In conclusion, the results highlight the importance of certain items, such as A6 and A7, in the assessment of adverse experiences, as well as the internal organization of the instrument. This analysis significantly contributes to validating and optimizing the ACE-ASF in the Ecuadorian context.

**Figure 3:**
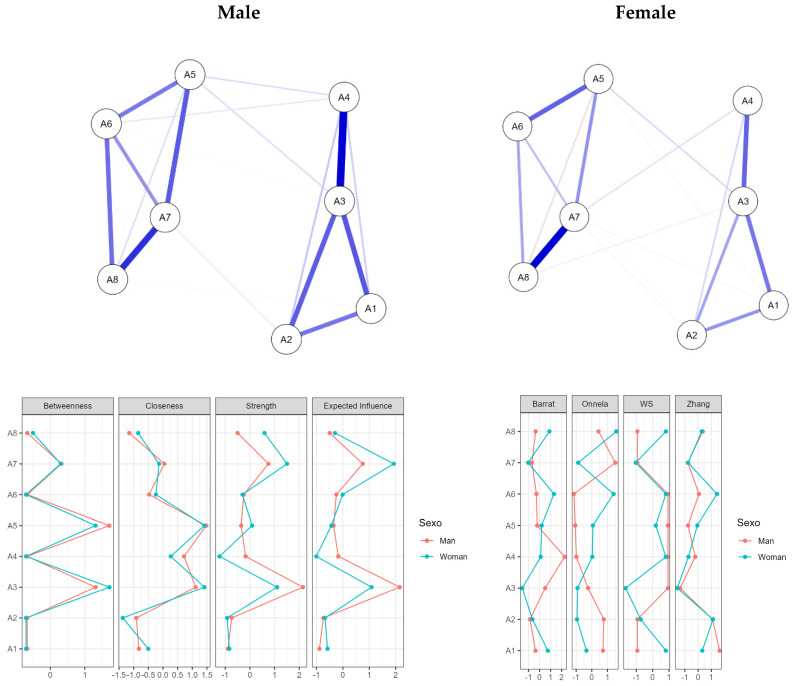
Networks, centrality, and clustering of the Adverse Childhood Experiences Abuse Short Form (ACE-ASF) in Ecuadorian youth as a function of sex.

Figure 3 presents the networks, centrality measures, and clustering analysis of the Adverse Childhood Experiences Abuse Short Form (ACE-ASF) in Ecuadorian youth, differentiated by sex (male and female). In the lower part of the figure, four centrality measures for the items (betweenness, closeness, strength, and expected influence) and four clustering methods (Barrat, Onnela, WS, and Zhang) are analyzed, comparing the results by sex. Regarding the centrality measures, “betweenness” shows differences in regards to how certain items act as intermediaries in the male and female networks. Closeness indicates the proximity of the nodes to the rest of the network, with variations depending on gender. Strength reflects the intensity of the connections, showing that females present weaker relationships compared to males. Finally, expected influence measures the overall influence of each node in the network, where some items indicated a greater influence in males.

Regarding the clustering methods, each technique (Barrat, Onnela, WS, and Zhang) identifies unique patterns regarding how the items cluster within the male and female networks. These analyses evidence clear differences in the structural organization of adverse experiences by sex, reflecting particular dynamics concerning how these experiences are processed or experienced. In summary, the figure highlights significant differences in the characteristics and structures of adverse experience networks between males and females. These differences suggest important implications for the design of gender-specific interventions and future research in the Ecuadorian context.

The network analysis of the Adverse Childhood Experiences Abuse Short Form (ACE-ASF) among Ecuadorian youth reveals a dense structure with eight nodes and 23 edges, indicating a sparsity of 17.9% (Supplementary Table S1). Centrality measures show that A3 and A5 are key nodes, with high betweenness, closeness, and strength, while A7 shows a high expected influence (Supplementary Table S2). The clustering analysis reveals A6’s strong presence in the subgroups, whereas A2 and A4 show weaker connections (Supplementary Table S3). The weight matrix highlights notable connections, i.e., A3–A4 (0.488) and A7–A8 (0.794), alongside weaker relationships, such as A5–A8 (−0.189) (Supplementary Table S4). The analysis also revealed gender differences: for both males and females, the network included eight nodes and 18 edges, resulting in a sparsity of 0.357 (Supplementary Table S5). Centrality measures indicated that A3 was central for both sexes, but more dominant for males. The importance of A5 and A7 varied by sex (Supplementary Table S6). Clustering measures differed by gender, with A4 exhibiting high clustering in males and A6 in females (Supplementary Table S7). The weight matrix revealed that the strongest link in males was between A3 and A4 (0.484), while in females, it was between A7 and A8 (0.7), suggesting that the connections between adverse experiences may differ based on gender (Supplementary Table S8).

The analysis of physical and emotional violence, as well as sexual violence, revealed gender differences in the reported scores. Females displayed a slightly higher mean score for physical and emotional violence (M = 11.500, SD = 3.427) compared to that of males (M = 11.100, SD = 3.625), with a more pronounced difference in regards to sexual violence (M = 8.868, SD = 2.630) compared to the results for males (*M* = 8.147, *SD* = 1.623). The median scores were similar for sexual violence across genders (*Med* = 8), while for physical and emotional violence, females scored higher (*Med* = 11) than males (*Med* = 10). The dispersion of data was lower for sexual violence among males, as indicated by its lower coefficient of variation (0.199). The Mann–Whitney U test showed that the difference in physical and emotional violence was marginally significant (*U* = 81,225.000, *p* = 0.051), whereas the difference in sexual violence was highly significant (*U* = 74,258.500, *p* < 0.001), with females scoring higher. The effect size, represented by the rank–biserial correlation, was small for physical and emotional violence (*r* = 0.077, *SE* = 0.040) but larger for sexual violence (*r* = 0.157, *SE* = 0.040), suggesting that gender differences were more pronounced in the latter dimension (Table 3).

## 4. Discussion

The main objective of the research was to analyze the psychometric properties of the abbreviated version of the Adverse Childhood Experiences questionnaire (ACE-ASF) in Ecuadorian youth, with a particular focus on ensuring its reliability, validity, and measurement invariance.

### 4.1. Reliability

The findings confirm that the ACE-ASF exhibits a high level of internal consistency in the Ecuadorian youth population, supporting its reliability as a tool for measuring adverse experiences. The Cronbach’s alpha coefficients (α = 0.823 for Factor 1: physical and emotional abuse; α = 0.867 for Factor 2: sexual abuse) reflect a strong association between the items and the constructs being evaluated. These results align with previous studies, such as those by Chegeni et al. (2020) in Iran and Meinck et al. (2017) in Romania, which also identified a bifactorial structure in the ACE-ASF.

Additionally, the inclusion of composite reliability (CR) in this research represents a significant advancement in the psychometric evaluation of the ACE-ASF, allowing for the measurement of the internal consistency of the model from a more comprehensive perspective. Unlike other traditional indicators, such as Cronbach’s alpha (Revelle, 2019), CR takes into account the factor loadings of the items, providing a more accurate estimate of the reliability of the construct. The results obtained in this study show CR values greater than 0.70, confirming that the scale demonstrates high internal coherence (Ramírez et al., 2025). This finding not only validates the applicability of the ACE-ASF in the Ecuadorian context but also establishes a solid foundation for its use in future studies on adverse childhood experiences.

### 4.2. Validity

The results of the present study confirm the validity of the original two-factor structure of the Adverse Childhood Experiences Abuse Short Form (ACE-ASF) proposed by Meinck et al. (2017) in a sample of Ecuadorian youth. The original model demonstrated excellent fit, evidenced by a non-significant Chi-square (*Χ*^2^ *=* 19.587, *p* = 0.420), a high comparative fit index (*CFI* = 0.999), a low root mean square error of approximation (*RMSEA* = 0.006, *CI:* 0.000–0.031), and a standardized root mean square residual (*SRMR* = 0.041). These results are consistent with previous research supporting the robustness of this factorial structure in diverse cultural contexts. The findings reinforce the applicability of the ACE-ASF for assessing childhood abuse experiences in Ecuadorian youth and provide a reliable foundation for its use in future research focused on this population.

The ACE-ASF demonstrated adequate convergent and discriminant validity, evidenced by the clear and precise differentiation between the dimensions of physical–emotional abuse and sexual abuse. The standardized factor loadings ranged from 0.707 to 0.832 for Factor 1 and from 0.654 to 0.858 for Factor 2, confirming the instrument’s capacity to measure specific constructs. These results are consistent with international research employing similar tools, such as the ACE-IQ, to assess adverse experiences in diverse cultural contexts (Almuneef et al., 2014; Goodman et al., 2017; Kidman et al., 2019; Soares et al., 2016). Studies like that of Xu et al. (2023) also emphasize the importance of refined scoring methodologies, which could benefit both the ACE-ASF and the ACE-IQ, particularly in regards to epidemiological and clinical research.

Additionally, the analysis of the variance inflation factor (VIF) was incorporated to assess the multicollinearity between the items of the scale, ensuring that there is no excessive redundancy that could affect the interpretation of the results (Kalnins & Praitis Hill, 2025). The VIF values obtained in this research were below the critical threshold of 10, indicating acceptable levels of independence between the items and strengthening the validity of the model. This indicator is crucial in the validation of the ACE-ASF, as it ensures that the observed relationships between the variables are genuine and not the result of artificially inflated correlations. Thus, a more solid and reliable structure is ensured, establishing a methodological standard for future psychometric research.

### 4.3. Measurement Invariance

The invariance tests by sex support the robustness and generalizability of the ACE-ASF. The results indicate that the configural, metric, scalar, and strict models maintained excellent fit indices, with CFI values consistently at 0.999, RMSEA at 0.000, and SRMR below 0.06. This evidence confirms that the ACE-ASF measures the underlying constructs equivalently for both males and females, ensuring that the comparisons between sexes are meaningful and free from bias. In addition to validating the use of the ACE-ASF in Ecuador, these findings set a precedent for its application in sex-based analyses in similar sociocultural contexts, thereby expanding its relevance and utility in the global study of adverse childhood experiences.

The ACE-ASF model was found to be gender-invariant, ensuring that the measurements are equivalent for both males and females. This finding is crucial for its applicability across various subgroups within the Ecuadorian population, particularly in research and clinical settings. Contrary to previous claims, the study by Meinck et al. (2017) included a sex invariance analysis. Their research established configural invariance, with fit indices suggesting that the factor structure of the ACE-ASF is equivalent for males and females (χ^2^ = 59.83). Subsequently, the metric model showed a comparable fit to the configural model (DIFFTEST Δχ^2^ (df) = 1.74(6), *p* = 0.942), allowing metric invariance to be assumed. However, when evaluating scalar invariance, the model fit deteriorated significantly compared to that of the metric model (DIFFTEST Δχ^2^ (df) = 101.62(14), *p* < 0.001), leading to the rejection of scalar invariance. These results highlight the importance of assessing sex invariance in the ACE-ASF and suggest that while factor loadings are equivalent between groups, differences in response thresholds may exist and should be considered in future studies. In this regard, the study by Casas Muñoz et al. (2024) in Mexico underscores the relevance of such analyses for identifying differential patterns in exposure to adverse experiences, emphasizing the need for further research examining these differences across diverse sociocultural contexts.

### 4.4. Comparisons with the ACE-IQ

In contrast to the ACE-ASF’s specific focus on abuse dimensions, the ACE-IQ offers a broader perspective by considering a variety of childhood adversities. Studies conducted in countries such as Kenya, Brazil, Saudi Arabia, and Iraq (Almuneef et al., 2014; Goodman et al., 2017; Soares et al., 2016) have demonstrated its utility, though they also highlight limitations regarding its psychometric validation, particularly in low-income settings (Kidman et al., 2019). The results from Ecuador for the ACE-ASF are comparable to those of these studies, particularly for identifying correlations between adverse experiences and mental health issues, such as depression and PTSD (Goodman et al., 2017; Soares et al., 2016). For example, the ACE-ASF in Ecuador revealed moderate correlations between the abuse dimensions (r = 0.299, *p* < 0.001).

In contrast, the ACE-IQ, with its broader scope, provides insights into a wider range of adverse experiences (Xu et al., 2023). The ACE-ASF, although more limited in focus, offers more specific and validated insights into the relationship between abuse and mental health in the Ecuadorian youth population. These differences suggest important implications for the design of gender-specific interventions, the development of longitudinal studies, and future research in the Ecuadorian context.

## 5. Conclusions

The Ecuadorian experience with the ACE-ASF underscores the need for cultural adaptations to maximize the validity and reliability of these tools. Specifically, cultural biases and stigmatization associated with the sexual abuse dimension presented significant challenges in regards to data collection. This finding aligns with the results of studies such as that of Chen et al. (2022), which highlighted the influence of scoring methodologies and cultural barriers on the interpretation of results. Both the ACE-ASF and the ACE-IQ are valuable tools for assessing adverse experiences in diverse cultural contexts. In Ecuador, the ACE-ASF demonstrated its utility by providing reliable and culturally sensitive measurements of physical–emotional and sexual abuse. At the same time, international studies on the ACE-IQ highlight its capacity to assess a broader spectrum of childhood adversities. Integrating both perspectives can enrich our understanding of adverse experiences and their impact on mental health, facilitating the design of targeted and culturally adapted interventions.

The Adverse Childhood Experiences Abuse Short Form (ACE-ASF) is a reliable and suitable tool for assessing childhood adversities among Ecuadorian youth. Its factorial structure, consisting of two dimensions (physical and emotional abuse and sexual abuse), aligns with previous findings in international contexts such as Iran and Romania. Reliability analyses showed satisfactory values, and sex invariance confirmed its applicability for both males and females, allowing for equitable comparisons. However, the high skewness and kurtosis observed in items related to sexual abuse suggest that cultural and social factors may influence the willingness to report such experiences, highlighting the importance of considering context when interpreting results.

Despite its significance, this study presents certain limitations. The sample used was specific and non-probabilistic, limiting the generalizability of the findings to other populations in the country. Additionally, the use of self-report questionnaires may be subject to biases such as selective recall or social desirability, particularly regarding sensitive topics like sexual abuse. The cross-sectional design prevents the establishment of causal relationships between adverse experiences and their impact on psychological well-being. Furthermore, sociodemographic variables such as socioeconomic status or ethnicity were not included, which could have provided valuable insights for a more comprehensive analysis.

Future research should address these limitations by expanding the sample to include diverse regions and populations within Ecuador, including children, adolescents, and older adults. It would also be beneficial to explore how cultural and social factors influence the perception and reporting of adverse experiences, particularly sexual abuse, to adapt the instrument to local contexts. Longitudinal studies would allow for a deeper understanding of the long-term effects of these experiences on mental health and psychological development. Additionally, incorporating variables such as resilience, social support, and mental health could help identify factors that moderate or mediate the impact of childhood adversities. Finally, comparing findings with those from other Latin American countries would strengthen the cross-cultural validity of the ACE-ASF and contribute to the development of prevention and intervention programs aimed at mitigating the negative effects of childhood adversities.

## Figures and Tables

**Table 1:** Descriptions of the items and dimensions of the Adverse Childhood Experiences Abuse Short Form (ACE-ASF) in Ecuadorian youth (*n* = 840).

	Median	Mean	SD	Skewness	Kurtosis	*p*	Q1	Q3
A1	3	3.283	1.219	−0.159	−1.133	<0.001	2	4
A2	2	2.512	0.980	1.109	0.503	<0.001	2	3
A3	3	3.025	1.162	0.285	−1.050	<0.001	2	4
A4	2	2.491	0.988	1.119	0.469	<0.001	2	3
A5	2	2.242	0.775	1.601	3.213	<0.001	2	2
A6	2	2.117	0.646	1.769	5.340	<0.001	2	2
A7	2	2.096	0.609	2.106	7.914	<0.001	2	2
A8	2	2.070	0.595	2.259	8.944	<0.001	2	2

**Note:** SD = std. deviation, *p* = *p*-value of Shapiro–Wilk test, Q1 = 25th percentile, and Q3 = 75th percentile.

**Table 2:** Loadings, reliability, and validity of the original model of the Adverse Childhood Experiences Abuse Short Form (ACE-ASF) in Ecuadorian youth.

Factor	Indicator	Std. Est. (All)	α	ω	CR	VIF	ω Total	α Total	F1	F2
F1	A1	0.707	0.823	0.83	0.83	1.42	0.872	0.812	0.553 *	
	A2	0.705								
	A3	0.832								
	A4	0.705								
F2	A5	0.858	0.867	0.861	0.85	1.56			0.367 **	0.637 *
	A6	0.805								
	A7	0.816								
	A8	0.654								

**Note.** F1 = physical and emotional abuse, F2 = sexual abuse, * = AVE (convergent validity), bold ** = HTMT (discriminant validity), ω = McDonald’s omega, α = Cronbach’s alpha, CF = composite reliability, and VIF = variance inflation factor.

**Table 3:** Differences in the dimensions of the Adverse Childhood Experiences Abuse Short Form (ACE-ASF) by sex.

	Physical and Emotional		Sexual	
	Male	Female	Male	Female
*n*	402	438	402	438
Mean (M)	11.100	11.500	8.147	8.868
Std. deviation	3.625	3.427	1.623	2.630
Median (Med)	10	11	8	8
IQR	6	5	0	1
Minimum	4	4	4	4
Maximum	20	20	18	20
25th percentile	8	9	8	8
50th percentile	10	11	8	8
75th percentile	14	14	8	9
SE	0.181	0.164	0.081	0.126
Coefficient of variation	0.327	0.298	0.199	0.297
Mean rank	403,552	436,055	386,223	451,960
Sum rank	162,228.000	190,992.000	155,261.500	197,958.500
*U*	81,225.000		74,258.500	
*p*	0.051		<0.001	
VS-MPR *	2.430		10,461.152	
Rank–Biserial correlation (*r*)	0.077		0.157	
SE Rank–Biserial correlation	0.040		0.040	

**Note:** VS-MPR * = Vovk-Sellke Maximum p-Ratio, *U* = Mann–Whitney U.

## Data Availability

The data supporting this research are publicly available and can be obtained by emailing the first author of this article.

## Supplementary Materials

The following supporting information can be downloaded at: https://www.mdpi.com/article/10.3390/ejihpe15040063/s1.

## Abbreviations

The following abbreviations are used in this manuscript:

ACE	Adverse Childhood Experiences Questionnaire
ACE-ASF	Adverse Childhood Experiences Abuse Short Form
AVE	average variance extracted
HTMT	heterotrait–monotrait ratio
CR	composite reliability
VIF	variance inflation factor
CFI	comparative ft index
TLI:	Tucker–Lewis index
GFI	goodness of fit index
RMSEA	root mean square error of approximation
SRMR	standardized root mean square residual

## Appendix A

### Appendix A.1

The reliability analysis of the Adverse Childhood Experiences Abuse Short Form (ACE-ASF) applied to Ecuadorian youth (*n* = 840) showed robust results (Table A1). For the total scale (items A1–A8), the lambda-2 coefficient (*λ2*) had an estimated value of 0.835, while the lambda 6 (*λ6*) reached 0.867, and the greatest lower bound (*GLB*) was 0.906. These indicators suggested high internal consistency for the complete scale. The average inter-item correlation (*AIC*) was reported as 0.376, with a total mean score of 19.831 (*SD* = 4.741). Additionally, the 95% confidence intervals indicated stable estimates, with lower bounds of 0.813, 0.844, and 0.890, respectively, and upper bounds of 0.854, 0.887, and 0.921. When analyzing specific dimensions, the first dimension (F1, physical and emotional abuse, items A1–A4) obtained an estimated λ2 of 0.826, a λ6 of 0.788, and a *GLB* of 0.860, with an *AIC* of 0.540. The average scores for this dimension were 11.308 (*SD* = 3.527), indicating high internal consistency. The 95% confidence intervals were also narrow, ranging from 0.803 to 0.845 for *λ2*, 0.763 to 0.811 for *λ6*, and 0.837 to 0.880 for *GLB*. In the second dimension (F2, sexual abuse, items A5–A8), the reliability estimates were even higher. The *λ2* showed an estimated value of 0.869, *λ6* reached 0.853, and *GLB* was 0.914, with an AIC of 0.633. The average scores for this dimension were 8.523 (*SD* = 2.234). The confidence intervals for *λ2* ranged from 0.834 to 0.898, for *λ6* from 0.814 to 0.888, and for *GLB* from 0.885 to 0.937, demonstrating the stability of these measures. In conclusion, the results supported the reliability of the ACE-ASF instrument for assessing adverse childhood experiences in the Ecuadorian population, both for the total scale and for its specific dimensions.

**Table A1:** Reliability statistics of the Adverse Childhood Experiences Abuse Short Form (ACE-ASF) in Ecuadorian youth (*n* = 840).

	Estimate	*λ*2	*λ*6	*GLB*	*AIC*	Mean	*SD*
*Total*	Point estimate	0.835	0.867	0.906	0.376	19.831	4.741
(A1–A8)	95% CI lower bound	0.813	0.844	0.890	0.332	19.510	4.524
	95% CI upper bound	0.854	0.887	0.921	0.416	20.152	4.979
*F1*	Point estimate	0.826	0.788	0.860	0.540	11.308	3.527
(A1–A4)	95% CI lower bound	0.803	0.763	0.837	0.501	11.070	3.366
	95% CI upper bound	0.845	0.811	0.880	0.573	11.547	3.704
*F1*	Point estimate	0.869	0.853	0.914	0.633	8.523	2.234
(A5–A8)	95% CI lower bound	0.834	0.814	0.885	0.565	8.372	2.132
	95% CI upper bound	0.898	0.888	0.937	0.697	8.674	2.346

**Note.** *F1* = physical and emotional abuse, *F2* = sexual abuse, *λ2* = Guttman’s *λ2*, *λ6* = Guttman’s *λ6*, *GLB* = greatest lower bound, and *AIC* = average interitem correlation.

### Appendix A.2

The factor analysis indicated that the results of both the Kaiser–Meyer–Olkin (*KMO*) index and Bartlett’s test of sphericity were satisfactory in all cases, suggesting the adequacy of the data for conducting a factor analysis (Table A2). Regarding the *KMO* index, the observed values for each item (A1 to A8) were above 0.7, indicating a good sample adequacy. In the general analysis, the *KMO* was 0.808, reflecting an appropriate suitability for the analysis. Specifically, the *KMO* values for males and females were very similar, with males achieving a value of 0.792 and females 0.810, further supporting the robustness of the analysis for both genders.

The *R*^2^, which measures the variance explained by each item, ranged from 0.317 to 0.797, with items A5, A6, and A7 showing the highest explained variance. Ther results of the Bartlett’s test of sphericity, which tests the null hypothesis that the correlation matrix is an identity matrix, were significant for all three groups (general, males, and females), with *p*-values < 0.001. This suggests that the correlations between items were strong enough to justify the use of a factor analysis. In conclusion, the results showed that the data were appropriate for conducting a factor analysis, with *KMO* indices and Bartlett’s test of sphericity results supporting the validity of the analyses for both the general group and the subgroups of males and females.

**Table A2:** Kaiser–Meyer–Olkin and Bartlett’s test results for factor analysis by group.

	General		Male		Female	
	*KMO*	*R* ^2^	*KMO*	*R* ^2^	*KMO*	*R* ^2^
A1	0.818	0.499	0.816	0.513	0.813	0.483
A2	0.865	0.496	0.841	0.504	0.872	0.474
A3	0.771	0.692	0.773	0.709	0.775	0.684
A4	0.830	0.497	0.821	0.515	0.845	0.484
A5	0.815	0.736	0.802	0.537	0.811	0.782
A6	0.834	0.648	0.795	0.453	0.833	0.719
A7	0.788	0.666	0.751	0.559	0.798	0.797
A8	0.768	0.427	0.736	0.317	0.771	0.590
Overall	0.808		0.792		0.810	
	Bartlett’s test of sphericity					
*Χ* ^2^	3.099.719		1.242.090		1.729.997	
*df*	28		28		28	
*p*	<0.001		<0.001		<0.001	

**Note.** *KMO* = Kaiser–Meyer–Olkin test; *R*^2^ *= R-squared.*
