# Supplementary material for: Psychometric Properties of the Adverse Childhood Experiences Abuse Short Form (ACE-ASF) for Ecuadorian Youth
Source: Eur J Investig Health Psychol Educ. 2025 Apr 16;15(4):63. doi: 10.3390/ejihpe15040063 (PMC12025786; doi:10.3390/ejihpe15040063)
Supplement: Supplementary file 1 [file ejihpe-15-00063-s001.zip › ejihpe-3482306-supplementary.pdf]

Supplements

In Supplementary Table S1, the network structure was detailed, including eight nodes and 23 of the 28 possible edges, reflecting a sparsity of 17.9%. These results indicated that the network was relatively dense, suggesting strong interconnections between the items in the questionnaire.

Supplement Table S1. *Summary of Network of the Adverse Childhood Abuse Experiences Short Form (ACE-ASF) in Ecuadorian youth as a function of sex.*

Number of nodes	*Number of non-zero edges*	*Sparsity*
8	23 / 28	0.179

In Supplementary Table S2, the centrality measures of the network items were reported. Items A3 and A5 stood out for having high values in betweenness, closeness, and strength, indicating that they played a key role in connecting other nodes within the network and facilitating information flow. In contrast, items A1, A2, A4, and A6 showed negative values in most metrics, reflecting a lower relative importance within the network structure. On the other hand, item A7 exhibited high expected influence, suggesting that it contributed significantly to the overall impact of the network.

Supplement Table S2. *Centrality measures per variable*

	Network			
Variable	*Betweenness*	*Closeness*	*Strength*	*Expected influence*
A1	-0.553	-0.524	-0.872	-0.533
A2	-0.553	-1.037	-0.812	-0.627
A3	2.028	1.620	1.099	1.308
A4	-0.553	-0.345	-1.160	-0.680
A5	1.106	1.518	0.445	-0.654
A6	-0.553	-0.511	-0.794	-0.444
A7	-0.369	-0.160	1.061	1.863
A8	-0.553	-0.561	1.034	-0.234

Supplementary Table S3 presented the clustering measures of the items. Item A6 stood out for having high clustering values according to *Barrat* and *Zhang's* methods, indicating that it belonged to well-defined subgroups within the network. Conversely, items A2 and A4 had lower values, suggesting weaker connections with specific clusters.

Supplement Table S3. *Clustering measures per variable*

	**Network**			
Variable	Barrat	Onnela	WS	Zhang
A7	-0.605	0.982	-1.260	-0.404
A8	-1.021	0.304	0.900	-0.077
A1	0.986	-0.986	0.900	0.623
A2	-0.767	0.341	-1.260	0.967
A3	0.882	0.623	0.900	-2.159
A4	-0.191	-1.873	-0.540	-0.056
A5	-0.860	0.927	-0.540	0.212
A6	1.575	-0.318	0.900	0.893

Finally, Supplementary Table S4 showed the weight matrix of the connections between items. The strongest relationships were observed between A3 and A4 (*weight* = 0.488) and between A7 and A8 (*weight* = 0.794). These relationships highlighted the direct interaction between certain items that assessed similar dimensions of adverse experiences. In contrast, weak or negative relationships were observed between items such as A5 and A8 (*weight* = -0.189), reflecting less significant or inverse interactions. In conclusion, the network analysis identified important patterns in the interconnection and relevance of the ACE-ASF items among Ecuadorian youth. These findings provided valuable insights to enhance the interpretation of the questionnaire and its application in future research or interventions.

Supplement Table S4. *Weights matrix*

	Network							
Variable	A1	A2	A3	A4	A5	A6	A7	A8
A1	0.000	0.299	0.362	0.018	0.054	-0.024	0.000	0.020
A2	0.299	0.000	0.287	0.103	-0.044	0.000	0.061	0.000
A3	0.362	0.287	0.000	0.488	0.104	0.041	0.000	-0.077
A4	0.018	0.103	0.488	0.000	0.000	0.031	0.040	0.012
A5	0.054	-0.044	0.104	0.000	0.000	0.389	0.385	-0.189
A6	-0.024	0.000	0.041	0.031	0.389	0.000	0.068	0.247
A7	0.000	0.061	0.000	0.040	0.385	0.068	0.000	0.794
A8	0.020	0.000	-0.077	0.012	-0.189	0.247	0.794	0.000

The network analysis of the Adverse Childhood Experiences Short Form (ACE-ASF) in Ecuadorian youth revealed significant differences based on sex. The network for both men and women consisted of eight nodes and 18 non-zero edges out of a total possible 28, resulting in identical sparsity of 0.357 (Supplement Table S5).

Supplement Table S5. *Summary of Network of the Adverse Childhood Abuse Experiences Short Form (ACE-ASF) in Ecuadorian youth as a function of sex.*

Network	*Number of nodes*	*Number of non-zero edges*	*Sparsity*
Man	8	18 / 28	0.357
Woman	8	18 / 28	0.357

Regarding centrality measures, the results highlighted that, in men, variable A3 showed the highest values for strength, closeness, and expected influence, indicating its crucial role within the network. Similarly, in women, A3 also held a central position, though with less dominance compared to men. Other variables, such as A5 and A7, also demonstrated variable importance between the sexes, depending on the metric used (Supplement Table S6).

Supplement Table S6. *Centrality measures per variable*

	Man				Woman			
Variable	Betweenness	Closeness	Strength	Expected influence	Betweenness	Closeness	Strength	Expected influence
A1	-0.670	-0.823	-0.890	-0.890	-0.706	-0.502	-0.834	-0.581
A2	-0.670	-0.910	-0.738	-0.738	-0.706	-1.372	-0.925	-0.679
A3	1.314	1.118	2.152	2.152	1.715	1.415	1.101	1.087
A4	-0.670	0.719	-0.174	-0.174	-0.706	0.267	-1.231	-1.007
A5	1.711	1.475	-0.355	-0.355	1.311	1.415	0.089	-0.437
A6	-0.670	-0.474	-0.248	-0.248	-0.706	-0.242	-0.301	-0.011
A7	0.322	0.046	0.753	0.753	0.303	-0.134	1.507	1.929
A8	-0.670	-1.151	-0.499	-0.499	-0.504	-0.845	0.593	-0.302

Clustering measures revealed differentiated patterns between men and women. In men, A4 showed high levels of clustering according to Barrat's metric, whereas in women, this variable had comparatively lower values. Conversely, A6 displayed a higher level of clustering in the female network, especially according to Zhang's metric, which might reflect differences in how certain experiences are interrelated depending on sex (Supplement Table S7).

Supplement Table S7. *Clustering measures per variable*

	Man				Woman			
Variable	Barrat	Onnela	WS	Zhang	Barrat	Onnela	WS	Zhang
A7	-0.691	1.507	-0.935	-0.775	-1.031	-0.870	-1.039	-0.754
A8	-0.354	0.430	-0.935	0.343	0.888	1.567	0.808	0.268
A1	-0.369	0.714	-0.935	1.617	0.751	-0.341	0.808	0.310
A2	-0.862	0.768	-0.935	1.091	-0.646	-0.943	-0.731	1.116
A3	0.506	-0.236	0.935	-1.357	-1.558	-0.911	-1.655	-1.572
A4	2.267	-0.985	0.935	-0.221	0.106	0.030	0.808	-0.727
A5	-0.215	-1.059	0.935	-0.761	0.189	0.073	0.192	-0.060
A6	-0.281	-1.138	0.935	0.063	1.301	1.395	0.808	1.419

The weight matrix revealed the strongest connections between variables. In men, the most significant relationship was found between A3 and A4, with a weight of 0.484. In women, the most prominent connection was between A7 and A8, with a weight of 0.7. These differences suggested that the links between certain adverse experiences could vary considerably by gender (Supplement Table S8). Overall, the results underscored important differences in the structure and characteristics of the networks between men and women.

These distinctions could reflect variations in how adverse experiences in childhood are perceived or experienced, emphasizing the importance of considering sex in studies related to this type of experience.

Supplement Table S8. *Weights matrix*

				Man				
Items	A1	A2	A3	A4	A5	A6	A7	A8
A1	0	0.26	0.322	0.1	0	0	0	0.01
A2	0.26	0	0.307	0.11	0	0	0.04	0
A3	0.32	0.31	0	0.48	0.069	0	0	0
A4	0.1	0.11	0.484	0	0.071	0.05	0	0
A5	0	0	0.069	0.07	0	0.26	0.31	0.069
A6	0	0	0.003	0.05	0.257	0	0.21	0.276
A7	0	0.04	0	0	0.312	0.21	0	0.399
A8	0.01	0	0	0	0.069	0.28	0.4	0
				**Woman**				
Items	A1	A2	A3	A4	A5	A6	A7	A8
A1	0	0.3	0.379	0	0.027	0	0.03	0
A2	0.3	0	0.254	0.12	0	0.01	0.03	0
A3	0.38	0.25	0	0.43	0.109	0	0	-0.05
A4	0	0.12	0.428	0	0	0	0.09	0
A5	0.03	0	0.109	0	0	0.43	0.3	-0.1
A6	0	0.01	0	0	0.434	0	0.18	0.247
A7	0.03	0.03	0	0.09	0.296	0.18	0	0.699
A8	0	0	-0.05	0	-0.1	0.25	0.7	0

Supplement Table S9. Original two-factor model and sex invariance of the Adverse Childhood Experiences Abuse Short Form (ACE-ASF) in Ecuadorian youth

Models	X^2	*df*	*p*	*CFI*	*RMSEA*	*CI RMSEA*	*SRMR*
Original model	19,587	19	0.420	.999	0.006	0.000 - 0.031	0.041
**Invariance by Sex**							
Configural	27,058	38	0.907	.999	0.000	0.000 - 0.014	0.047
Metric	30,927	44	0.932	.999	0.000	0.000 - 0.010	0.045
Scalar	42,399	50	0.769	.999	0.000	0.000 - 0.023	0.049
Strict	49,101	58	0.791	.999	0.000	0.000 - 0.021	0.057

Note: Original model of eight items by Meinck et al. [42].

Supplement Table S10. Reliability and validity (convergent and discriminant) of the gender invariance of the Adverse Childhood Experiences Abuse Short Form (ACE-ASF) in Ecuadorian youth

Invariance testing	Group		Factor 1	Factor 2	ω	α	ω Total	α Total
Configural	Male	Factor 1	0.588*		0.848	0.842	0.857	0.775
		Factor 2	**0.227****	0.531*	0.794	0.801		
	Female	Factor 1	0.524*		0.812	0.804	0.878	0.833
		Factor 2	**0.458****	0.673*	0.880	0.886		
Metric	Male	Factor 1	0.588*		0.848	0.842	0.852	0.775
		Factor 2	**0.227****	0.514*	0.797	0.801		
	Female	Factor 1	0.522*		0.811	0.804	0.877	0.833
		Factor 2	**0.458****	0.675*	0.877	0.886		
Scalar	Male	Factor 1	0.588*		0.847	0.842	0.852	0.775
		Factor 2	**0.227****	0.515*	0.796	0.801		
	Female	Factor 1	0.522*		0.811	0.804	0.877	0.833
		Factor 2	**0.458****	0.675*	0.877	0.886		
Strict	Male	Factor 1	0.578*		0.834	0.842	0.837	0.775
		Factor 2	**0.227****	0.496*	0.771	0.801		
	Female	Factor 1	0.530*		0.823	0.804	0.890	0.833
		Factor 2	**0.458****	0.695*	0.907	0.886		

Note: Factor 1 = Physical and Emotional Abuse, Factor 2 = Sexual Abuse, * =AVE (Convergent validity), Bold ** = HTMT (Discriminant validity), ω = Omega de McDonald, α = alfa de Cronbach.

Supplement Figure S1. Physical and Emotional by Sex

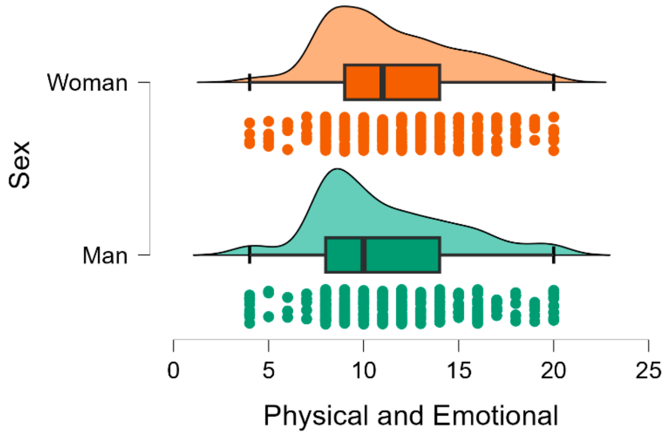

Supplement Figure S2. Sexual by Sex

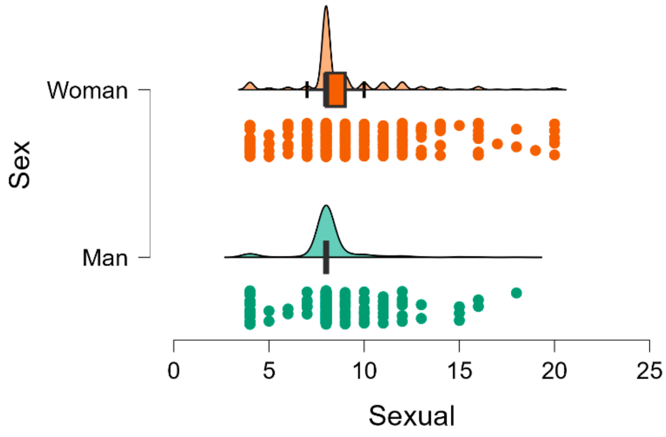

Supplement Figure S3. Raincloud Plots the Physical and Emotional Sex and age

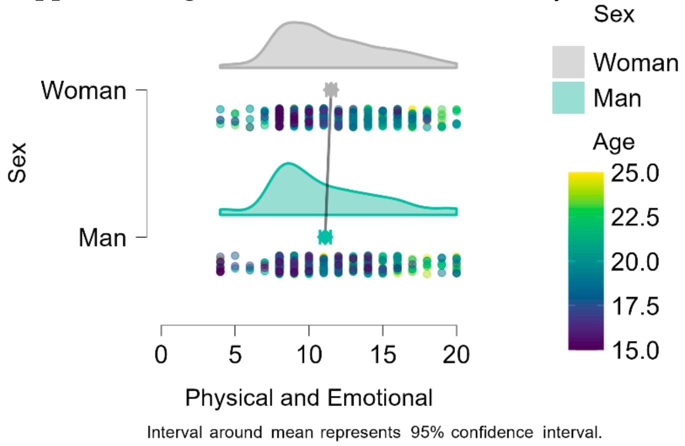

Supplement Figure S4. Raincloud Plots the Sexual by Sex and age

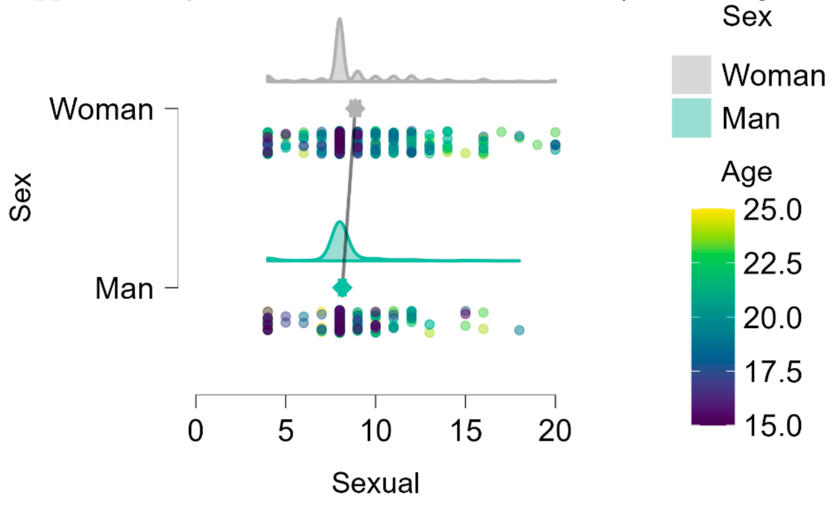

Interval around mean represents 95% confidence interval.
